# Groove pancreatitis, diagnosis, and management strategies: a case series

**DOI:** 10.1093/jscr/rjag238

**Published:** 2026-04-11

**Authors:** Ahmed Aydrose, Adnan Abdalla, Mouaz Idris, Hussein Emad, Mujtaba Hageldaw, Salah Aldeen A Sulieman

**Affiliations:** Department of Surgery, University Hospital Limerick, St. Nessan's Road, Dooradoyle, Limerick, V94 F863, Munster, Ireland; Department of Surgery, University Hospital Limerick, St. Nessan's Road, Dooradoyle, Limerick, V94 F863, Munster, Ireland; Department of Surgery, University Hospital Limerick, St. Nessan's Road, Dooradoyle, Limerick, V94 F863, Munster, Ireland; Department of Surgery, University Hospital Limerick, St. Nessan's Road, Dooradoyle, Limerick, V94 F863, Munster, Ireland; Department of Surgery, National Ribat University, 61 Street, Burri Al-Sharif, Khartoum 11111, Sudan; Department of Surgery, National Ribat University, 61 Street, Burri Al-Sharif, Khartoum 11111, Sudan

**Keywords:** groove pancreatitis, para-duodenal pancreatitis, pancreatic adenocarcinoma

## Abstract

Groove pancreatitis (GP) is identified as an inflammatory process that entails the anatomical part that lies between the C-loop of the duodenum and the head of the pancreas as well as common bile duct. Diagnosis is often challenging due to the extensive similarities with pancreatic adenocarcinoma; therefore, histological examination is needed for a proper differentiation. We report a case series of two male patients with a history of alcoholism who presented with upper abdominal pain, nausea, and vomiting. Computed tomography revealed inflammatory changes around the head of the pancreas and duodenum, indicating GP. Conservative management formed the mainstay of treatment, with surgical intervention reserved for intractable symptoms and complications. Both patients responded well and were referred to hepatobiliary surgeons for further management. Early recognition is crucial to avoid misdiagnosis and to prevent unnecessary major surgery, underscoring the need for a detailed history, careful imaging interpretation, and multidisciplinary evaluation.

## Introduction

Groove pancreatitis (GP) is a rare subtype of chronic pancreatitis affecting the pancreaticoduodenal groove. It is most commonly seen in men in their 50s and 60s and is strongly associated with chronic alcohol consumption, tobacco use, ulcers of the gallbladder, gastrectomy, and biliary tract disease. This disease is localized in the duodenal groove which is an anatomical gap existing between the pancreas and duodenum. Differentiation of GP and pancreatic adenocarcinoma should be accurately determined to make correct diagnoses of pancreatic head masses or duodenal stenosis [1, 2].

The first description of the condition was by Becker in 1973 as ‘Rinnenpankreatitis’ and later termed ‘groove pancreatitis’ by Stolte *et al.* in 1982. GP accounts for 2.7%–24.5% of pancreaticoduodenectomies performed for chronic pancreatitis [3–5]. Advances in imaging have improved preoperative diagnosis, facilitating early management, and reducing misdiagnoses [6].

## Cases series

### Case 1

We present a case of 33-year-old male, initially diagnosed with pancreatitis in 2007. In 2024, the patient presented with acute epigastric pain radiating to the back associated with nausea and vomiting. First, the patient denied alcohol or smoking history. Through physical examination revealed tenderness on the epigastric area. Blood investigations revealed elevated inflammatory markers and a raised serum amylase level of 266 U/L (normal range: 28–100), while liver enzymes, including bilirubin, were within normal limits.

The patient has experienced recurrent episodes of pancreatitis over the past 8 years, with similar attacks of severe epigastric pain. Abdominal ultrasound and magnetic resonance cholangiopancreatography (MRCP) ruled out cholelithiasis and choledocholithiasis consistently. Despite thorough investigations, including endoscopy and endoscopic ultrasound done in 2018, no significant inflammatory or malignant features were identified. As a result, the underlying etiology of the recurrent pancreatitis remains elusive.

Hypertriglyceridemia was determined during the last admission with 10.3 mmol/L (normal level: 0.31.7) being considered as the possible causative factor of the condition. However, when a detailed history was taken, excess alcohol intake, which has not been mentioned previously, has been uncovered.

**Figure 1:**
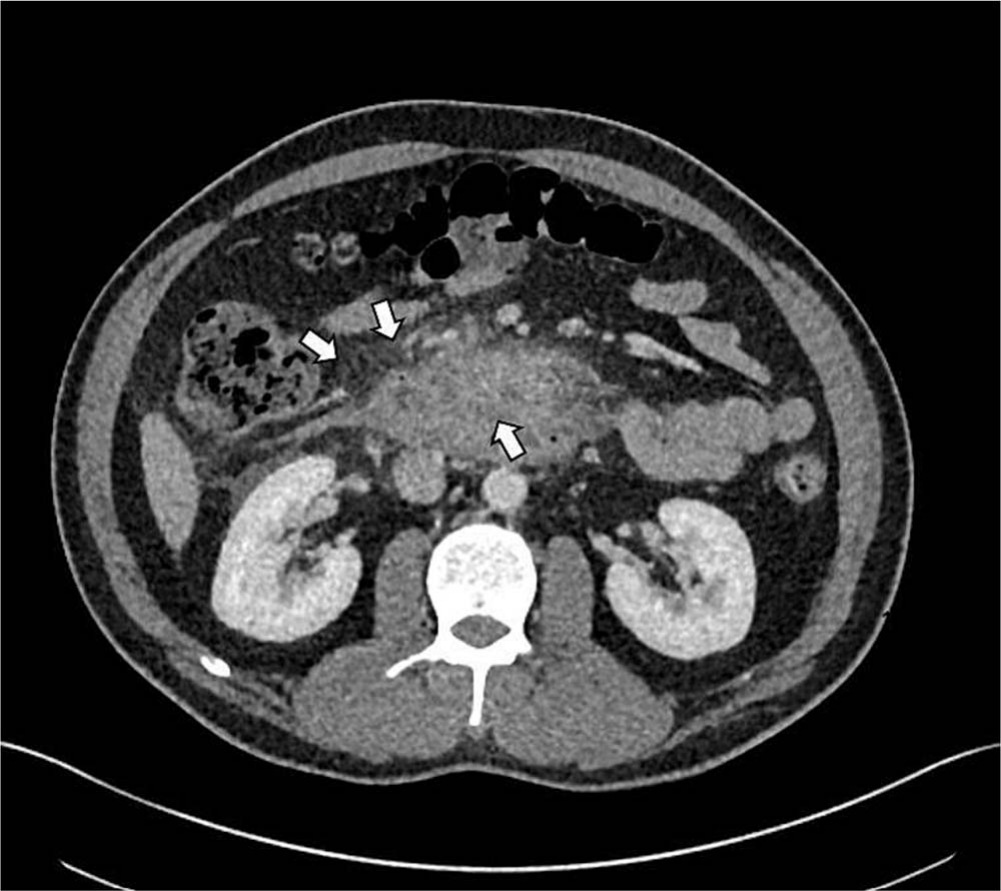
Shows axial CT scan showing prominent reactive mesenteric lymphadenopathy and mucosal oedema in the second and third parts of the duodenum (*indicated by arrows*), consistent with reactive inflammation in GP.

Abdominal and computed tomography of the abdomen and the pelvis (CTAP) showed oedematous change in the pancreatic head, uncinate process and also featured peripancreatic fat stranding which was deepened into the anterior pararenal spaces which were mostly right sided. There were no focal necrosis and homogeneous enhancement in the pancreas. Additionally, there was prominent reactive mesenteric lymphadenopathy and mucosal oedema in the second and third parts of the duodenum, likely reactive in nature (Fig. 1).

**Figure 2:**
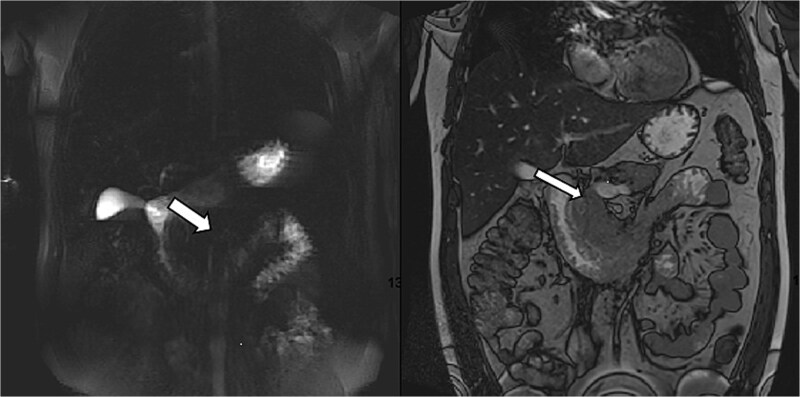
MRCP demonstrating features suggestive of GP. Arrows indicate the inflammatory changes and associated duodenitis in the pancreaticoduodenal groove.

MRCP ruled out cholelithiasis and choledocholithiasis. However, it demonstrated features suggestive of GP with associated duodenitis (Fig. 2).

### Case 2

A 52-year-old male, a known case of Crohn’s disease and a long history of alcohol misuse, presented to the emergency department with two-ay history of central abdominal pain associated with nausea and vomiting. He had no known history of similar symptoms.

On examination, the patient had tenderness over the epigastric region. Additionally, a reducible right inguinal swelling was observed, indicating an uncomplicated inguinal hernia. Blood investigations showed high inflammatory markers, a serum amylase level of 208 U/L (normal range: 28–100), and high liver enzymes, including alkaline phosphatase at 153 U/L (normal range: 40–129), gamma-glutamyl transferase at 817 U/L (normal range: 10–71), and alanine aminotransferase at 76 U/L (normal range: 10–50). Also, his bilirubin was elevated at 28 μmol/L (normal range: 3–21). Urine amylase was also elevated at 586 U/L (normal range: 16–491).

We admitted the patient in the ward and commenced supportive treatment in form of intravenous fluids, analgesia, and antibiotics.

**Figure 3:**
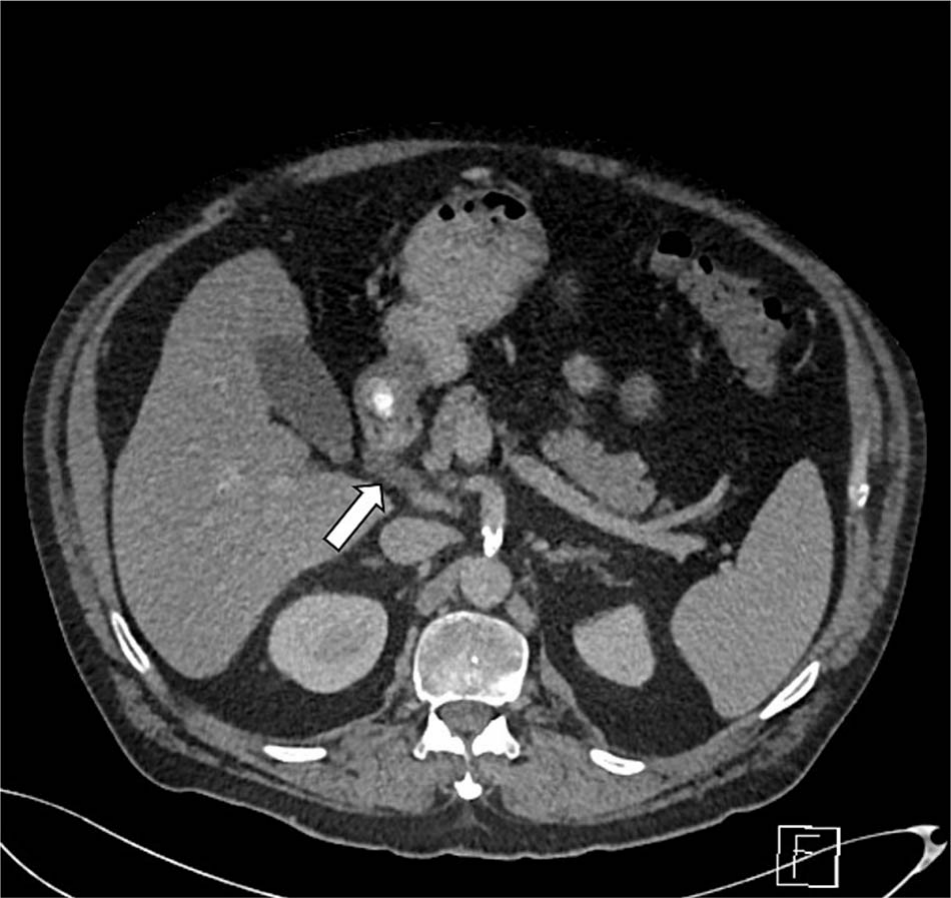
Axial section of abdominal CT scan. The axial section of the abdominal CT scan shows inflammatory fat stranding around the head and uncinate process of the pancreas.

**Figure 4:**
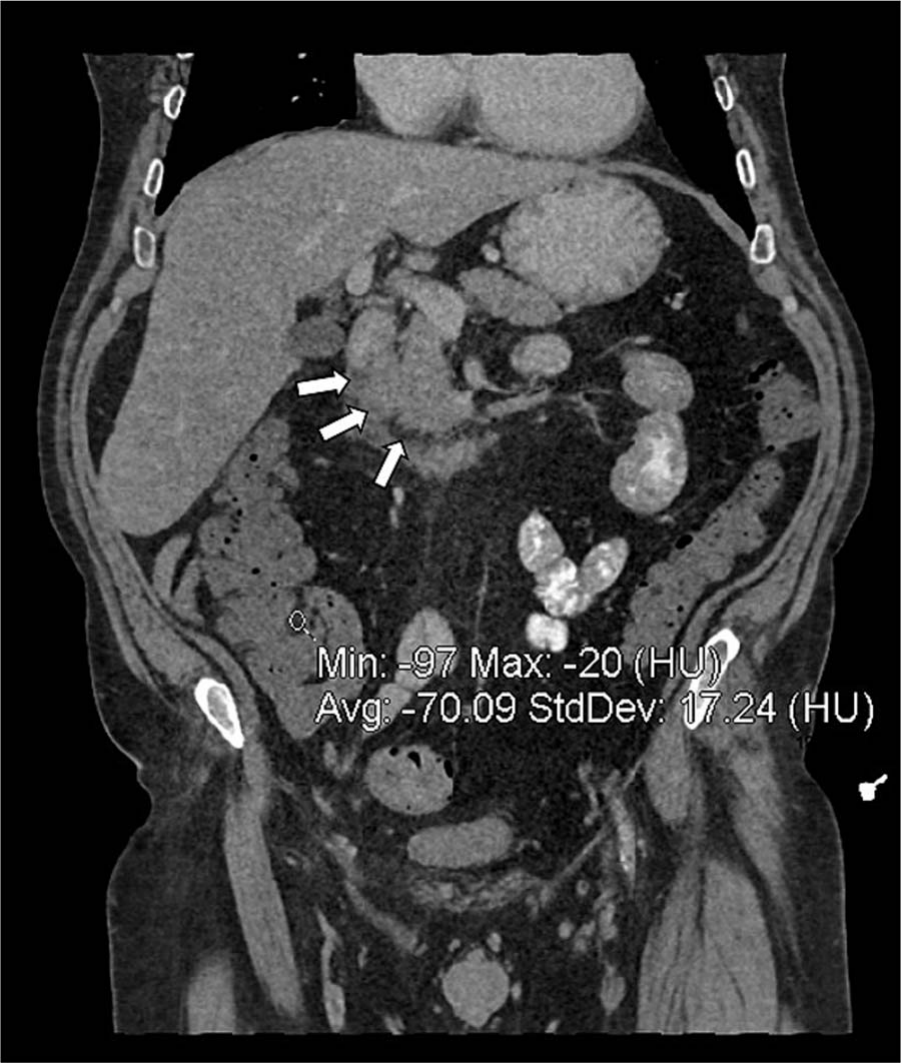
Coronal reconstruction of abdominal CT showing inflammatory fat stranding (arrows) in the pancreaticoduodenal groove, between the duodenum and the uncinate process of the pancreas.

**Figure 5:**
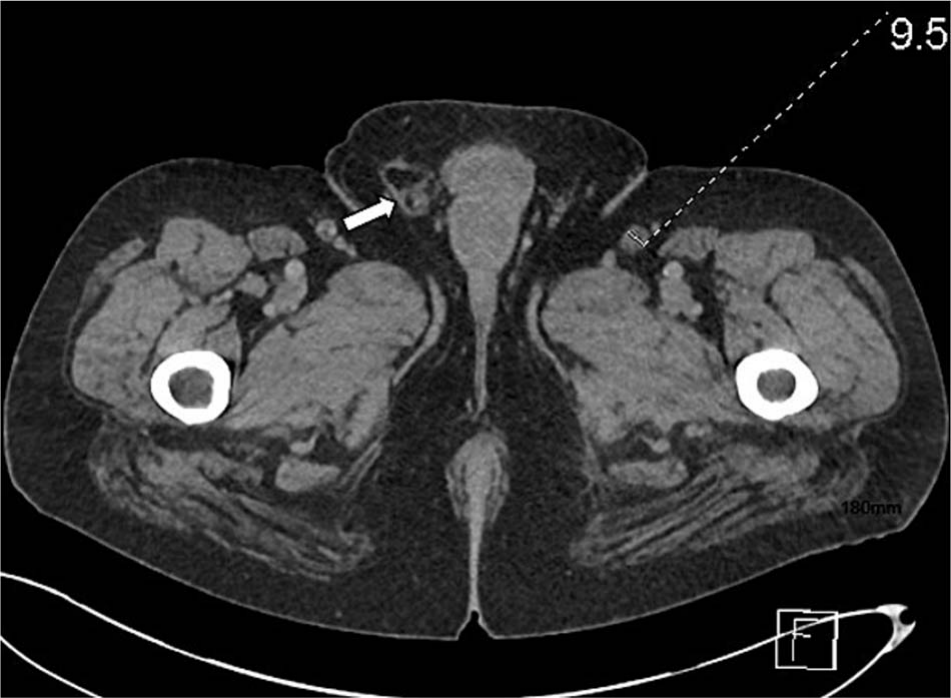
Axial section of abdominal CT scan. The axial section of the abdominal CT scan shows a right sided fat containing inguinal hernia.

CTAP showed focal inflammatory fat stranding and minor un-encapsulated fluid around and between the second and proximal third parts of the duodenum, with an eccentrically thickened medial wall. The inflammation extended to involve the head and uncinate process of the pancreas (Figs 3 and 4). Acute groove-type pancreatitis is the favored diagnosis given the elevated amylase levels. However, acute duodenitis remains a differential diagnosis. Additionally, mural fat in the caecum and ascending colon was observed, consistent with chronic Crohn’s disease (Fig. 4), and a right-sided fat-containing inguinal hernia was also noted (Fig. 5).

The radiographic characteristics of GP include a hypodense lesion that is localized to the head of the pancreas in close relation to the duodenal wall, thickening of the duodenal wall, which is related to mucosal oedema, and reactive mesenteric lymphadenopathy. The key components of the differential diagnosis include pancreatic adenocarcinoma, which is generally presented in the form of an irregular mass with signs of vascular invasion and concomitant double-duct dilation, and duodenal adenocarcinoma, which is manifested by a focus of thickening of the duodenal wall and possible pancreatic invasion and local lymphadenopathy. Additionally, periampullary carcinoma is characterized by a small enhancing lesion accompanied by biliary dilatation.

GP was differentiated from pancreatic adenocarcinoma by the presence of a relatively smooth, well-defined inflammatory mass in the pancreaticoduodenal groove, with less aggressive ductal distortion and absence of clear vascular encasement on contrast-enhanced computed tomography (CT). MRCP demonstrated groove-localized inflammation and duodenitis without a focally enhancing mass or typical ‘double-duct’ sign suggestive of pancreatic head malignancy (Figs 1–3).

Both patients were admitted under general surgery and subsequently reviewed at a multidisciplinary team meeting. Supportive treatment was commenced, in form of intravenous fluids, broad-spectrum antibiotics, antiemetics, and Gemfibrozil (300 mg), as well as analgesia, proton pump inhibitors, and pancreatic enzyme supplementation. Vital parameters and clinical status were monitored closely throughout admission. Following stabilization and full optimization of their condition, a consultation with the hepatobiliary surgery team was arranged, and the patient was transferred to their care for further follow-up and management. For the second case gastroenterology team was consulted for further management and follow-up in regard to the Crohn’s disease.

Both patients recovered well following conservative management, and were discharged with Gemfibrozil, proton pump inhibitor, and pancreatic enzyme supplements as their pain completely settled and blood tests returned to normal. Second case had seen by gastroenterology team during his admission, and they arranged for further follow-up in their clinic. Both patients were referred to the hepatobilliary surgery team for further management. Additionally, both patients were referred to a support program for alcohol addiction management.

## Discussion

GP, was named Rinnenpankreatitis by Becker in 1973, and later was introduced as ‘groove pancreatitis’ by Stolte *et al.* in 1982. Stolte described it as a distinct form of segmental pancreatitis characterized by fibrous scarring within the anatomical space between the dorso-cranial portion of the pancreatic head, the duodenum, and the common bile duct. GP is categorized into two forms: the pure form, where scarring is localized to the groove area, and the segmental form, which extends into the dorso-cranial pancreatic head [3].

Despite the uncertainty around the exact etiology, GP is thought to be linked with chronic alcohol use, smoking, and pancreatic duct obstruction as well as Brunner gland hyperplasia, and ectopic pancreatic tissue. The main pathological mechanism appears to involve an anatomical or functional obstruction of the minor papilla [7, 8]. On the other hand, GP typically presents in males during their fifth or sixth decades of life and clinical features include recurrent episodes of upper abdominal pain, early satiety, nausea, vomiting, and weight loss, which are primarily attributed to duodenal stenosis [9]. Jaundice is rare in GP and often raises suspicion of an underlying malignancy [8].

Chronic pancreatitis (GP) and pancreatic adenocarcinoma of the head are entities that should be distinguished as both may appear as similar on imaging studies. Computed tomography and magnetic resonance imaging are however often unreliable because of the fibrosis and scarring that is highly prevalent in GP and can resemble malignancy [5].

Treatment and intervention of GP vary between conservative management to surgical management depending on the severity and course of the disease. The conservative treatment includes dietary changes, analgesic medication, pancreatic rest, and absolute alcohol and smoking abstinence that may be used on a short-term basis. Somatostatin analog, e.g. octreotide, is somewhat effective, however, enteral nutrition is often hampered by duodenal obstruction, sometimes requiring parenteral nutrition. Endoscopic treatments (ductal dilation and pseudocyst drainage) can be used in the early stages of the disease, but could be restricted due to more extensive scarring. In cases of refraction or in case malignancy has not been ruled out, surgery, including pancreaticoduodenectomy (the Whipple procedure), is recommended [1].

In our series, conservative management was indicated for patients with mild-to-moderate symptoms, preserved ductal patency, and no evidence of biliary or duodenal obstruction. This approach typically results in symptom control, although some patients may experience recurrent flares. In contrast, surgical management, including pancreaticoduodenectomy or segmental resection, is reserved for patients with refractory pain, progressive duodenal or biliary obstruction, or persistent suspicion of malignancy. Surgical intervention is associated with better long-term symptom relief in selected cases but carries higher morbidity, so an individualized approach is essential.

The presented case series points to the need to review and thoroughly update patient histories, particularly with regards to alcohol consumption, in cases of recurrent pancreatitis. The extended diagnosis of gallstone pancreatitis demonstrates the challenges associated with the diagnosis of rare forms of the disease. A multidisciplinary approach, including careful clinical history taking, the use of advanced diagnostic images and the most appropriate diagnostic methods is necessary in order to achieve accurate diagnosis and the correct therapeutics.

Although the conservative management often results in the effective treatment, surgical intervention is an essential option that may be offered to the chosen patients, and it is necessary to stress the necessity of considering the individual approach to the treatment.

## Conclusion

GP is uncommon and often under-observed disease. Therefore, increased awareness is crucial for prompt diagnosis, as delayed identification may result in extended symptoms and unsuitable treatment. It is vital to obtain a detailed history, including alcohol consumption, as this may establish significant etiological factors. Because GP and pancreatic cancer have quite similar characteristics; careful differential diagnosis work up is required. Effective management necessitate collaboration between different specialties.

## Conflicts of interest

None declared.

## Funding

None declared.

## Patients’ consents

Obtained.

## References

[1] Brar HS, Shah NJ, Bukeirat F. Groove Pancreatitis. In: Abosheaishaa H, Abu-Ghosh A, Acharya C, et al. (eds.), StatPearls. Treasure Island (FL): StatPearls Publishing; 2024.

[2] Jani B, Rzouq F, Saligram S et al. Groove pancreatitis: a rare form of chronic pancreatitis. N Am J Med Sci 2015;7:533–2. 10.4103/1947-2714.170624 26713302 PMC4683809

[3] Stolte M, Weiss W, Volkholz H et al. A special form of segmental pancreatitis: "groove pancreatitis". Hepatogastroenterology. 1982;29:198–208. 7173808

[4] Tezuka K, Makino T, Hirai I et al. Groove pancreatitis. Dig Surg 2010;27:149–52. 10.1159/000289099 20551662

[5] Kutty SA, Chirukandath R, Pj B et al. Groove pancreatitis: a case report and review of a hidden type of chronic pancreatitis. Cureus 2022;14:e27738. 10.7759/cureus.27738 36134063 PMC9481208

[6] Ray S, Ghatak S, Misra D et al. Groove pancreatitis: report of three cases with brief review of literature. Indian J Surg 2017;79:344–8. 10.1007/s12262-017-1643-x 28827910 PMC5549052

[7] Adsay NV, Zamboni G. Paraduodenal pancreatitis: a clinico-pathologically distinct entity unifying “cystic dystrophy of heterotopic pancreas,” “paraduodenal wall cyst,” and “groove pancreatitis”. Semin Diagn Pathol 2004;21:247–54. 10.1053/j.semdp.2005.07.005 16273943

[8] Kim JD, Han YS, Choi DL. Characteristic clinical and pathologic features for preoperative diagnosed groove pancreatitis. J Korean Surg Soc 2011;80:342–7. 10.4174/jkss.2011.80.5.342 22066058 PMC3204702

[9] Kager LM, Lekkerkerker SJ, Arvanitakis M et al. Outcomes after conservative, endoscopic, and surgical treatment of groove pancreatitis: a systematic review. J Clin Gastroenterol 2017;51:749–54. 10.1097/MCG.0000000000000746. 27875360

